# Effects of antibiotic prescribing for respiratory tract infection on future consultations in primary care: a systematic review and meta-analysis

**DOI:** 10.1136/bmjopen-2025-099357

**Published:** 2025-07-28

**Authors:** Ibrahim Adamu, Amanda Lambert, Safiyya Bello, Fatima Aminu Abdulmalik, Tom Marshall

**Affiliations:** 1 University of Birmingham, Birmingham, UK

**Keywords:** Primary Care, Antibiotics, Behavior

## Abstract

**Abstract:**

**Objectives:**

Prescribing antibiotics may reinforce patients’ beliefs that antibiotics are needed and increase future consultations for similar symptoms. This review determines the effect of antibiotic prescribing for respiratory infections in primary care on future reattendance.

**Design:**

A systematic review and meta-analysis of randomised controlled trials (RCTs) and cohort studies and reported following Preferred Reporting Items for Systematic Reviews and Meta-Analyses guidelines. Participants were adults or children presenting with respiratory infection in primary care.

**Data sources:**

MEDLINE (Ovid), PubMed, Embase, the Cochrane Central Register of Controlled Trials, clinical trial registries and grey literature sources were searched from inception until 6 February 2024.

**Eligibility criteria:**

Eligible studies included open-label RCTs or cohort studies of antibiotics compared with no antibiotics in adults or children with respiratory infections. The outcome of interest was reattendance at least 28 days after the initial consultation.

**Data extraction and synthesis:**

Two reviewers independently screened, selected, assessed the quality and extracted data. Separate meta-analyses were presented for RCT and cohort studies and a combined meta-analysis of all studies.

**Results:**

We identified 2128 records and reviewed 48 full texts, of which five met the inclusion criteria. These reported three RCTs (1207 randomised to antibiotics, 672 controls) and three cohort studies (209 138 exposed to antibiotics, 46 469 controls). In the meta-analysis of RCTs, relative risk (RR) of reattendance with antibiotics was 1.10 (95% CI: 0.99 to 1.23), and in cohort studies, RR was 1.21 (95% CI: 0.94 to 1.49). An important limitation is that most studies were in UK primary care.

**Conclusion:**

Evidence suggests prescribing antibiotics for acute respiratory tract infections in primary care probably modestly increases future reattendance for similar conditions. Reducing antibiotic prescribing may help decrease demand for primary care.

**PROSPERO registration number:**

CRD42023470731.

**Strengths and limitations of this study**

- This is the first systematic review and meta-analysis of the effects of antibiotic prescribing for upper respiratory tract infections (URTIs) on future reattendance for URTI.
- The systematic review used a comprehensive search for both randomised trials and cohort studies reporting the effects of antibiotics on reattendance.
- The strength of evidence was assessed using the Grading of Recommendation, Assessment, Development and Evaluation approach.
- Almost all studies were conducted in the UK, which may limit the generalisability.
- All studies were published more than a decade ago.

## Introduction

 Although upper and lower respiratory symptoms are common in the UK, over 90% of those reporting sore throat, cough, cold or influenza symptoms do not consult a general practitioner.^1 2^ Those who do consult are commonly prescribed antibiotics, and consultations for respiratory symptoms account for most antibiotic prescribing in primary care.^3^ Indeed, changing rates of antibiotic prescribing are mainly attributable to changing rates of consultation (patient behaviour) and less by changing rates of prescribing for those who consult (clinician behaviour).^4^ Consultations for respiratory symptoms also contribute substantially to primary care workload.^5^

Patient belief in the efficacy of the doctor’s care and in the efficacy of self-care are both strongly associated with the decision to consult.^6^ This suggests that when patients feel their complaint can be treated without consulting a doctor, they are less likely to consult. Patient expectations are also associated with antibiotic prescribing, although not as strongly as the doctor’s beliefs about those expectations.^7^ It may be possible for primary care practices to change patients’ beliefs and reduce consulting behaviours for respiratory conditions.^8^ Prescribing antibiotics for respiratory conditions may reinforce a patient’s belief that antibiotics are necessary for the condition and that they need to consult a doctor to obtain them, thus increasing future consultation rates for similar respiratory symptoms.^9^ If this medicalisation hypothesis is correct, prescribing antibiotics will increase future workload. The aim of this review is to assess the effects of prescribing antibiotics for respiratory infections in primary care on the frequency of future attendance for similar symptoms.

## Methods

A systematic review was conducted and was reported in line with the guidance of the Preferred Reporting Items for Systematic Reviews and Meta-Analyses.^10^

### Population, intervention (exposure), comparison, outcome and study types

Eligible studies included adults or children with respiratory tract infections in a primary care setting (the population of interest). Studies in secondary and tertiary care settings were excluded. Respiratory tract infections include upper respiratory infections (common cold, laryngitis, sore throat, pharyngitis or tonsillitis, acute rhinitis, acute rhinosinusitis and acute otitis media) and lower respiratory tract infections (LRTI) (cough, acute bronchitis, bronchiolitis, pneumonia and tracheitis).^11^ The intervention or exposure was the prescription of any antibiotic (immediate or delayed, any dose or for any duration), and the comparator was no-antibiotic prescription. The outcome of interest was reattendance for respiratory tract symptoms at least 4 weeks after the initial consultation. This was to avoid including short-term reattendance for unresolved symptoms.

Eligible studies included randomised controlled trials (RCTs), cohort studies and case-control studies with an open (unblinded) design. Patients must know if they received antibiotics for this to affect their beliefs in antibiotic effectiveness. Search terms specified a range of synonyms for upper respiratory tract infections (URTIs) and a range of synonyms for reattendance.

### Databases searched

The following databases were searched: MEDLINE OVID (1946 to 6 February 2024), PubMed (1966 to 6 February 2024), Embase (1974 to 6 February 2024), Cochrane Central Register of Controlled Trials (CENTRAL) and Web of Science (inception to 6 February 2024). (online supplemental tables 1–5). Clinicaltrials.gov and clinicaltrialsregister.eu were searched for ongoing or recently completed trials, and grey literature sources were searched (WHO, WHO website and the National Institute for Health and Care Excellence, NICE) were searched for relevant studies. Reference lists of the included studies and relevant systematic reviews were checked for pertinent further studies. Search strategies are included in online supplemental file 1.

### Screening and study selection

After the removal of duplicates, two independent reviewers (IA, SB or FA) screened titles and abstracts against the eligibility criteria. Disagreements were resolved in consultation with a third reviewer. Full texts were retrieved of eligible studies and studies where eligibility could not be determined. Two independent reviewers (IA and TM) assessed the retrieved full texts against the inclusion and exclusion criteria. Two independent reviewers appraised quality of included studies using the Cochrane Risk of Bias 2 tool for RCTs,^12^ the Newcastle–Ottawa scale for cohort and case-control studies.^13^ The certainty of evidence was assessed using the Grading of Recommendation, Assessment, Development and Evaluation (GRADE) criteria.^14^ Two independent reviewers extracted data from the included studies using a pre-specified data extraction form in Microsoft Excel (online supplemental tables 6 and 7). Records of excluded studies and reasons for exclusion were kept and presented. Disagreements on quality assessment or data extraction were resolved by discussion and with input from a third reviewer when consensus could not be reached.

### Meta-analysis

DerSimonian and Laird random effects meta-analyses were conducted due to expected heterogeneity between studies.^15^ The primary meta-analysis was conducted for RCTs. A second meta-analysis included RCTs and cohort studies, and a third included only cohort studies. Adjusted relative risks (RRs) were used where available. Results were presented as forest plots. The admetan package in Stata V.18 (StataCorp) was used for analysis and production of forest plots. Heterogeneity was assessed using χ^2^ and I^2^ statistics.^16^ Three-level random effects meta-analysis of studies using Stata package meta multilevel was conducted to account for non-independence of participants, for example where the same control group was used in different studies.^17^ Studies were nested within study designs. Three prespecified subgroup analyses were proposed: effect by study design (randomised controlled trials (RCTs) compared with cohort and case control studies); participant age (children compared with adults); the intervention type (immediate vs delayed antibiotics). If there were ten or more studies, publication bias was assessed using funnel plots.^18^ The review was registered on PROSPERO.^19^ Ethical approval was not sought as this is a review of secondary data sources.

### Deviations from protocol

The protocol specified a cut-off of 2 weeks to distinguish between short-term reattendance and long-term reattendance. After conducting initial searches, it was clear that most published studies used a cut-off of 4 weeks or 30 days. The definition of long-term reattendance was therefore modified to reflect this.

### Patient and public involvement

Patients and/or the public were not involved in the design, conduct, reporting or dissemination plans of the research.

## Results

**Figure 1:**
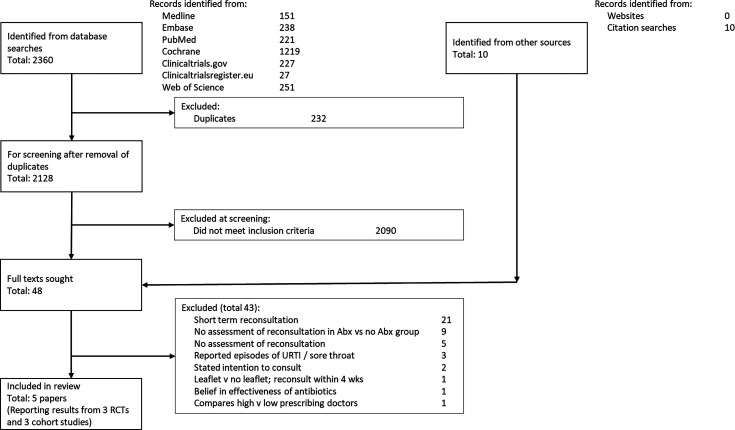
Preferred Reporting Items for Systematic Reviews and Meta-Analyses diagram of included studies. Abx, antibiotics; RCTs, randomised controlled trials; URTI, upper respiratory tract infections.

Database searches identified 2128 records after exclusion of duplicates. Of these, 38 met inclusion criteria, and a further 10 records were identified from citation searches. 48 full texts were reviewed, 43 were judged not to meet inclusion criteria, leaving five papers for inclusion (full texts included in online supplemental table 8). The primary reasons for exclusions were the outcome was short-term reattendance, relapse or non-resolution of symptoms (21); reattendance was not compared between groups receiving and not receiving antibiotics (9); reattendances were not reported (5); reported recurrent episodes of upper respiratory infections, rather than reattendances for URTI (3); reported intention to consult (2); reported belief in the effectiveness of antibiotics (1); the intervention was an information leaflet (1); and compared high and low-prescribing doctors (1). Some studies had more than one reason for exclusion (see figure 1).

The five papers reported findings from three RCTs and three cohort studies. One cohort study was an additional group of patients who were non-randomised and whose reattendances were reported in the same paper that reported findings of an RCT (Little 2014).^20^ RCTs included 1207 participants randomised to either immediate or delayed antibiotics and 672 to no antibiotics.^9 20 21^ Three cohort studies included 209 138 participants exposed to antibiotics and 46 469 unexposed controls^20 22 23^ (table 1). Williamson (2006)^23^ did not distinguish between short-term and long-term reattendance, but was included because over a long period of follow-up, it was assumed most reattendances would be after 28 days.

**Table 1:** Description of included studies

Study	Study design	Type of respiratory tract infection	Population	Country	Outcome	Intervention (antibiotic prescribing strategy)	Antibiotic arm: participants outcome/total	Control arm: participant outcome/total
Little *et al*^9^ 1997	RCT	Sore throat	Patients ≥4 years	England	Reattendance with sore throat 2 weeks to 1 year	Immediate prescription	106/222	78/192
						Delayed prescription	68/182	78/192
Moore *et al*^21^ 2009	RCT	LRTI	Patients ≥3 years	England	Reattendance with LRTI 1 to 12 months after the first visit	Immediate prescription	38/183	37/204
						Delayed prescription	37/188	37/204
Little *et al*^20^ 2014	RCT	Acute URTI and LRTI	Patients ≥3 years	UK	Reattendance with RTI after 1 month	Delayed: phone practice for prescription	42/107	39/122
						Delayed: post-dated prescription	45/114	39/122
						Delayed: collect prescription from practice	33/105	39/122
						Delayed: patient decides whether to use prescription	39/106	39/122
Herz *et al*^22^ 1988	Cohort study	Sore throat	Patients >21	Israel	Reattendance after 1 month	Immediate prescription	24/84	14/88
Williamson *et al*^23^ 2006	Cohort study	Acute otitis media	Any age	UK	Reattendance with acute otitis media at any time*	Antibiotics prescribed	83 987/208 728	16 992/46 259
Little *et al*^20^ 2014	Cohort study	Acute URTI and LRTI	Patients ≥3 years	UK	Reattendance with RTI after 1 month	Immediate prescription	136/326	39/122

* Did not distinguish between early and later reattendance.

LRTI, lower respiratory tract infection; RCT, randomised controlled trial; RTI, respiratory tract infection; URTI, upper respiratory tract infection.

### RCTs

Little 1997 compared outcomes with no antibiotics, delayed antibiotics or immediate antibiotics for sore throat but did not report reattendance rates by antibiotic strategy.^9^ Data were obtained after contacting the authors. Moore(2009) *et al*^21^ compared no antibiotics, delayed antibiotics or immediate antibiotics for acute LRTIs in a factorial design, which also evaluated the effects of providing a patient information leaflet.^21^ Little 2014 compared four delayed antibiotic strategies for acute respiratory tract infections: recontacting the practice by phone for a prescription, a post-dated prescription, allowing patients to collect the prescription themselves (collect) and prescribing but asking patients themselves to wait (patient led).^20^ The study also evaluated different strategies for symptom management in a factorial design and included a non-randomised comparison group of immediate antibiotics.

The RCTs were generally at low risk of bias (table 2). Selection of participants and randomisation processes were adequate; baseline characteristics were similar between allocated groups. There were relatively low rates of attrition and losses to follow-up, and outcome measurement and statistical analysis were appropriate. A change in the study protocol by Little *et al* 2014 to include an additional non-randomised arm was unlikely to have affected the findings. All three were conducted in UK primary care by the same group of UK researchers.

**Table 2:** Risk of bias assessment of the included studies

RCTs	Randomisation process	Deviations from intended intervention	Missing outcome data		Measurement of outcome		Selection of reported result			Overall risk of bias
Little *et al*^9^ 1997	Low risk	Low risk	Low risk		Low risk		Low risk			Low risk
Moore *et al*^21^ 2009	Low risk	Low risk	Low risk		Low risk		Low risk			Low risk
Little *et al*^20^ 2014	Low risk	Changes to protocol: unlikely to affect results	Low risk		Low risk		Low risk			Low risk
**Cohort studies**	**Selection**					**Comparability of cohorts**	**Outcome**			**Overall risk of bias**
	**Representativeness of the exposed cohort**	**Selection of non-exposed cohort**	**Ascertainment of exposure**	**Outcome of interest not present at baseline**			**Assessment of outcome**	**Duration of follow-up**	**Adequacy of follow-ups**	
Herz *et al*^22^ 1988	Low risk	Low risk	Low risk.	Not applicable		Limited information on differences between groups	Non-independent assessment	Low risk	Low risk	Moderate risk of bias
Williamson *et al*^23^ 2006	Low risk	Low risk	Low risk. Independently assessed from routine data	Not applicable		Differences between groups not described. Multivariable adjustment.	Low risk. Independently assessed from routine data	Low risk	Low risk	Low risk of bias
Little *et al*^20^ 2014	Low risk	Low risk	Low risk	Not applicable		Differences between groups. Multivariable adjustment.	Low risk	Low risk	Low risk	Low risk of bias

RCTs, randomised controlled trials.

### Cohort studies

A retrospective cohort study in a large database of UK primary care records compared reattendance rates in patients prescribed and not prescribed antibiotics for acute otitis media.^23^ Unadjusted hazard ratios and hazard ratios were adjusted for age, sex, multiple deprivation index, otorhinolaryngology referral and whether the patient was registered in high-prescribing general practice. Multivariable adjustment made only a small difference in HR, suggesting those prescribed and not prescribed antibiotics were broadly similar.

One of the cohort studies was a non-randomised group reported in the same paper as an RCT (Little 2014). The non-randomised group was prescribed immediate antibiotics and compared with the group who received no antibiotics. At baseline, these groups differed. The immediate antibiotics group had slightly more severe symptoms, was more likely to be labelled as LRTIs and less likely to be labelled as having URTIs. Findings were adjusted for symptom severity, symptom control strategies, smoking, prior infections, gender, age and duration of follow-up.

In a small interventional cohort study, a primary care clinician in Israel prescribed antibiotics to 50 sequential patients presenting with sore throats, did not prescribe antibiotics to the next 102 patients and prescribed to a final 50 patients. (Herz 1988)^22^ The paper reported reattendances 4 weeks to 6 months after the index consultation.^22^ Neither prescribing nor reattendance was independently assessed, and only limited information was provided on the comparability of the two groups.

All the cohort studies had low risk in the selection domain. For comparability, Little (2014) and Williamson (2006) scored two stars each, having adjusted for age and multiple other confounders. Herz (1988)^22^ scored zero, as it lacked baseline characteristics and did not adjust for confounders. Outcome assessment was independent in, Little (2014) and Williamson (2006), but not in Herz (1988).^22^ Overall, Little (2014) and Williamson (2006) were judged to have a low risk of bias, while Herz (1988)^22^ was rated moderate risk (table 2). Two of the three cohort studies were undertaken in the UK by the same group of UK researchers as the RCTs.

### Meta-analysis

**Figure 2:**
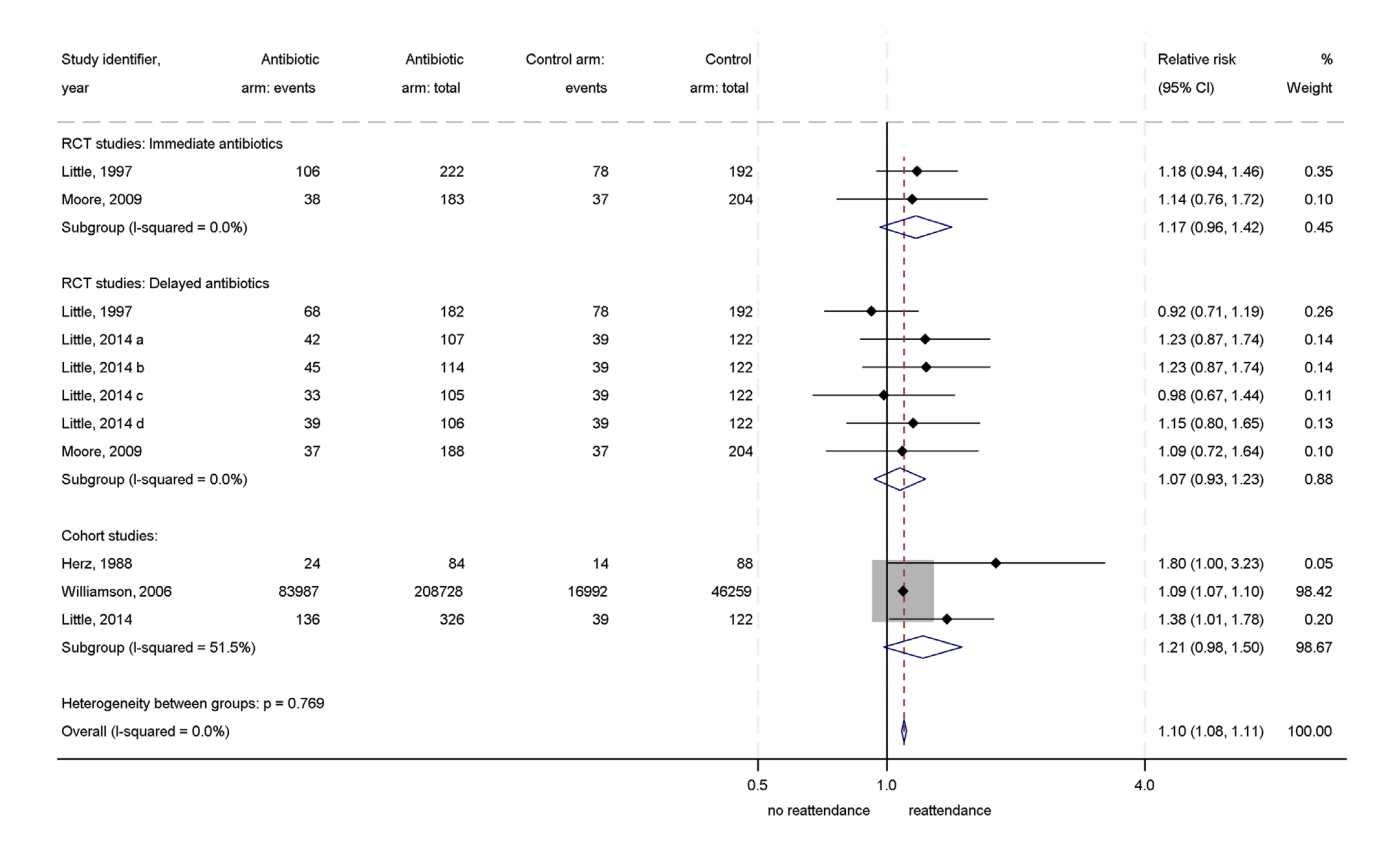
Forest plot of relative risk of reattendance for antibiotics vs no antibiotics, RCTs and cohort studies. RCT, randomised controlled trial.

**Figure 3:**
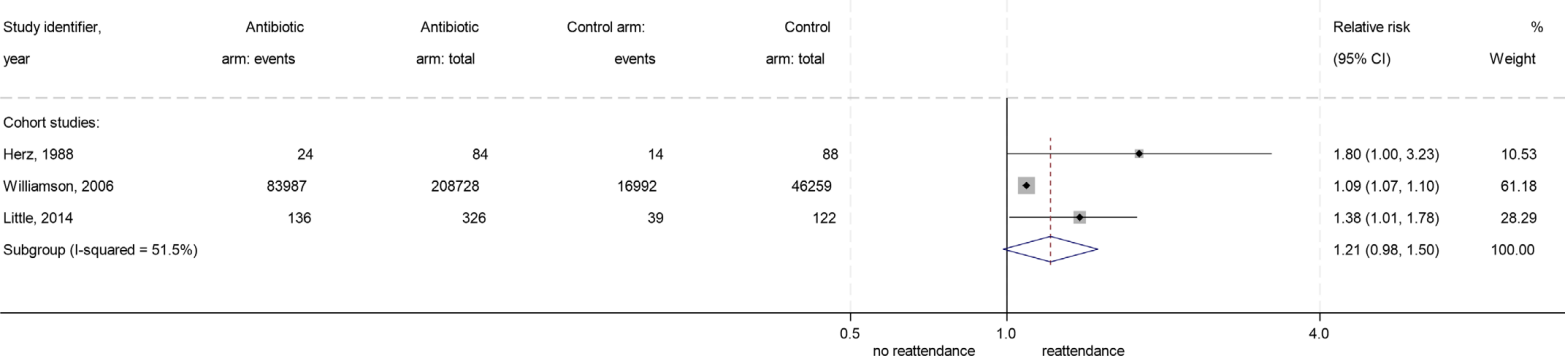
Forest plot of relative risk of reattendance for antibiotics vs no antibiotics, cohort studies.

In random effects meta-analysis of the RCTs, an RR of 1.10 (95% CI: 0.99 to 1.23) for reattendance was found in those randomised to antibiotics. There was no heterogeneity between included studies (I^2^=0%; τ^2^=0). Three of the RRs were calculated using the same control groups, thus were not independent. To test the effect of non-independence, a three-level random-intercepts meta-analysis of results within studies was carried out.^17^ This had no meaningful effect on the results (RR=1.10, 95% CI: 0.99 to 1.23) or on heterogeneity (I^2^=0%; τ^2^=0). Random effects meta-analysis of both RCTs and cohort studies found a combined RR of 1.10 (95% CI: 1.08 to 1.11) (figure 2). Three-level random effects meta-analysis found similar results (RR=1.09, 95% CI: 1.06 to 1.13). Random effects meta-analysis of cohort studies alone found an RR of reattendance in those receiving antibiotics of 1.21 (95% CI: 0.94 to 1.49), with moderate heterogeneity between the cohort studies (I^2^=51.5%, τ^2^=0.02) (figure 3).

**Figure 4:**
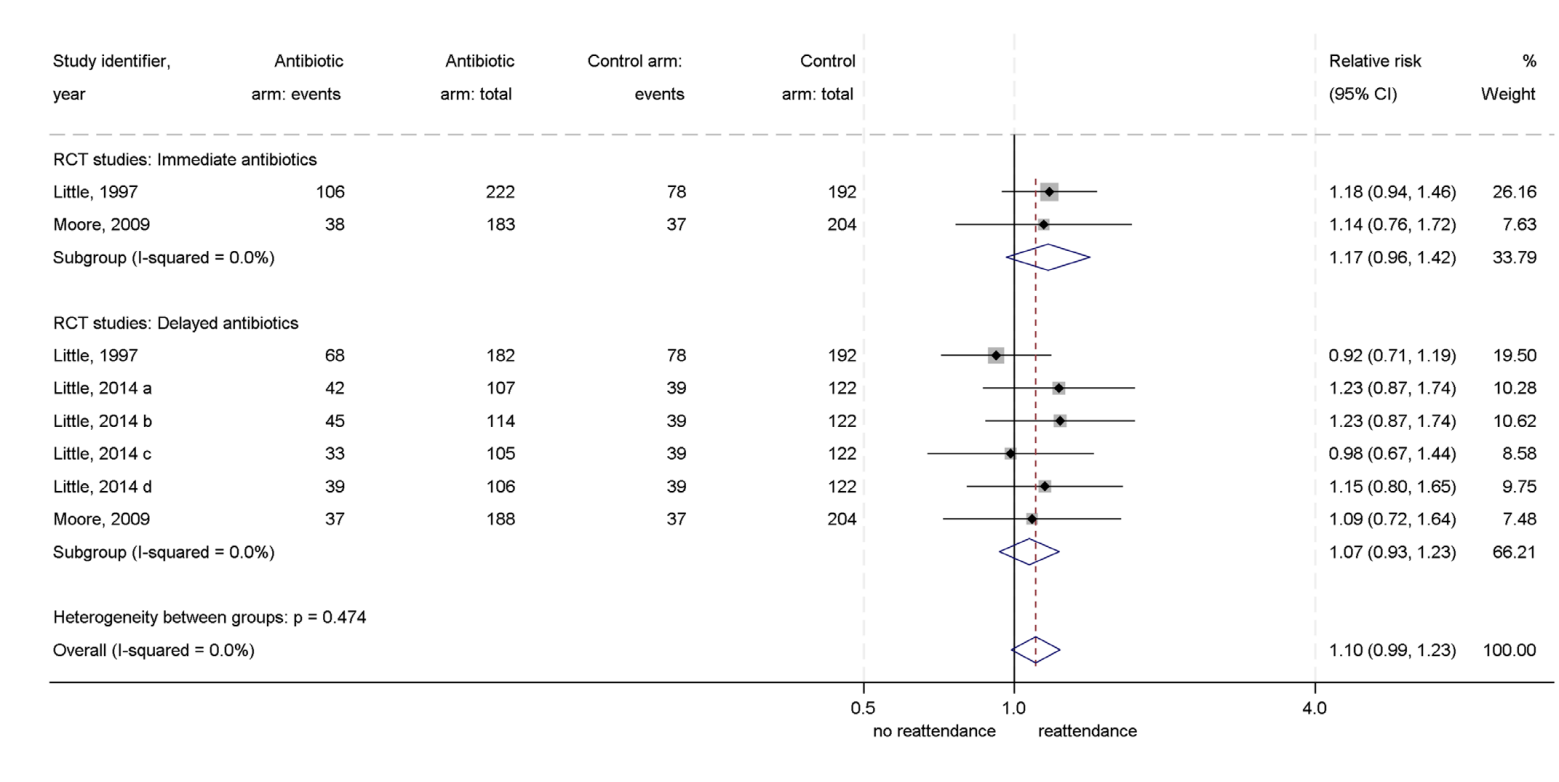
Forest plot of relative risk of reattendance for antibiotics versus no antibiotics: RCT, randomised controlled trial.

Subgroup analysis by the age of patients was not possible as all but one study reported combined results for patients of all ages. Similar results were found for immediate (RR=1.17, 95% CI: 0.92 to 1.42) and delayed (RR=1.07, 95% CI: 0.93 to 1.23) antibiotic prescribing strategies compared with no antibiotics (figure 4).

Because acute otitis media might be considered clinically different from the other conditions, further sensitivity analysis tested the effect of omitting the large acute otitis media cohort study (Williamson, 2006). This had little effect on the findings when RCTs and cohort studies were combined (RR=1.14, 95% CI: 1.03 to 1.27) (online supplemental figure 1) (RR=1.15, 95% CI: 1.04 to 1.28 in the three-level meta-analysis) but increased the effect in the cohort studies subgroup to 1.39 (95% CI: 1.07 to 1.80) (online supplemental figure 2).

## Discussion

### Summary

We found evidence that prescribing antibiotics for acute respiratory tract infections increases the frequency of reattendance for similar conditions.

### Strengths and limitations

Although RCT evidence alone did not reach statistical significance, the findings were consistent between RCT and cohort studies, across various respiratory infections, for both immediate and delayed antibiotic prescribing strategies. Applying the GRADE criteria, we rated the certainty of RCT evidence as high because of the consistency of the findings.^14^ The generalisability of the findings is less certain as almost all of the included studies were conducted in UK primary care, and all were conducted ten or more years ago. The cohort study evidence is at higher risk of bias than RCT evidence because of the possibility of unmeasured confounding, including confounding by indication, where antibiotics are prescribed to more severely affected patients. Nevertheless, both study types produced similar results.

### Comparison with existing literature

Other research has reached similar conclusions. A cohort analysis of 232 256 visits in 736 urgent care centres in the USA compared repeat consultation rates in patients consulting high-prescribing clinicians, of whom 81% prescribed antibiotics, to low-prescribing clinicians, of whom 42% prescribed antibiotics. Those consulting high-prescribing clinicians were more likely to reattend for acute respiratory infections: 68.8 vs 63.3 attendances per 100 (RR=1.09, p<0.001).^24^ The authors noted that assignment to clinician was ‘quasi-random’. An analysis of 108 UK general practices over a 6 year period found that general practices that reduce the proportion of acute respiratory infection consultations at which antibiotics are prescribed saw subsequent reductions in acute respiratory infection consultation rates.^25^

### Implications for research and practice

The ways in which antibiotic prescribing affects future prescribing behaviour may differ for different types of URTI. Prescribing antibiotics is hypothesised to affect patient belief in the effectiveness of antibiotics, intention to consult and therefore consulting behaviour. Several studies identified in the searches were excluded because they did not report consulting behaviour, but they found evidence of effects on belief and intention. Compared with no antibiotics, randomisation to immediate or delayed antibiotics increased the belief that antibiotics were effective for sore throat^26^ and for acute respiratory infections.^27^ A non-randomised comparison also found antibiotics increased belief in the effectiveness of antibiotics for acute respiratory infections.^20^ Random-effects meta-analysis gave a combined RR of 1.19 (95% CI: 0.86 to 1.64) for belief in antibiotic effectiveness. In one study, an immediate prescription for antibiotics (where 99% received antibiotics) also increased the belief that antibiotics were effective for acute otitis media compared with a delayed prescription (where 24% received antibiotics).^28^ Three of these trials also reported patients’ intentions to consult in the future were higher in those prescribed antibiotics.^26^,^28^ From clinical trials, we estimated the effects on reattendance of a single episode of prescribing (or not prescribing), but the effects of prescribing (or non-prescribing) over repeated consultations may have a cumulative effect on patients’ beliefs and therefore a potentially greater effect on reattendance. Medicalisation is not the only mechanism by which antibiotics might increase future consultation rates. Antibiotics may also increase the risk of clinical recurrence. Long-term follow-up of two double-blind RCTs of antibiotics in children found those assigned to antibiotics may have higher rates of carer-reported episodes of acute otitis media (RR: 1.46, 95% CI: 1.08 to 1.97) and sore throat (RR: 1.20, 95% CI: 0.80 to 1.78).^29 30^ Blinding eliminates the effect of carer beliefs about antibiotic effectiveness on carer-reported recurrences. Future research could investigate the contributions of beliefs and clinical recurrence to future consultation behaviour for different types of URTIs.

Despite the potential importance of prescribing as a cause of medicalisation, we found relatively few RCTs assessed longer-term changes in medicalisation or future consulting behaviour resulting from prescribing. This should be considered in all clinical trials of managing self-limiting illness in primary care. The predominance of evidence from UK primary care also points to a need for evidence from other settings. We did not address the question of whether immediate prescribing compared with delayed antibiotic prescribing has a similar effect on future consultation rates. This would require a search for studies with different intervention and comparison groups. The potential role of other types of discretionary prescribing on medicalisation and future consulting behaviour should also be explored.

### Implications for practice

It is plausible both that reducing antibiotic prescribing will decrease future consultations and that post-pandemic increases in antibiotic prescribing for upper respiratory infections may have increased demand.^31^ Practice policies that reduce antibiotic prescribing, such as dialogue with colleagues, consistent within-practice prescribing, supportive practice policies and increasing continuity of care may contribute to managing future demand for primary care.^32 33^

## Supplementary material

10.1136/bmjopen-2025-099357 online supplemental file 1

10.1136/bmjopen-2025-099357 online supplemental file 2

10.1136/bmjopen-2025-099357 online supplemental file 3

## Data Availability

All data relevant to the study are included in the article or uploaded as supplementary information.
