# Supplementary material for: Effects of antibiotic prescribing for respiratory tract infection on future consultations in primary care: a systematic review and meta-analysis
Source: BMJ Open. 2025 Jul 28;15(7):e099357. doi: 10.1136/bmjopen-2025-099357 (PMC12306365; doi:10.1136/bmjopen-2025-099357)
Supplement: online supplemental file 1 [file bmjopen-15-7-s001.pdf]

Ovid MEDLINE(R) ALL <1946 to February 6, 2024>

1 (respiratory tract adj3 infection$).ti,ab. 28086
2 (respiratory tract adj3 infection$).mp. [mp=title, book title, abstract, original title, name of substance word, subject heading word, floating sub-heading word, keyword heading word, organism supplementary concept word, protocol supplementary concept word, rare disease supplementary concept word, unique identifier, synonyms, population supplementary concept word, anatomy supplementary concept word] 60395
3 exp Pharyngitis/ 16776
4 acute pharyngitis.ti,ab. 640
5 exp Laryngitis/ 4136
6 acute laryngitis.ti,ab. 200
7 sore throat.mp. or Pharyngitis/ 13829
8 sore throat.ti,ab. 7115
9 exp Epiglottitis/ 1057
10 acute epiglottitis.ti,ab. 619
11 exp Tonsillitis/ 8264
12 acute tonsillitis.ti,ab. 783
13 exp Otitis Media/ 25856
14 acute otitis media.ti,ab. 5476
15 exp Otitis Externa/ 2668
16 acute otitis externa.ti,ab. 163
17 exp Sinusitis/ 23335
18 acute sinusitis.ti,ab. 1211
19 exp Rhinitis/ or exp Sinusitis/ 53552
20 acute rhinosinusitis.ti,ab. 686
21 exp Common Cold/ 4434
22 common cold.ti,ab. 4271
23 exp Tracheitis/ 1563
24 acute tracheitis.ti,ab. 13
25 exp Bronchitis/ 31580
26 acute bronchitis.ti,ab. 1540
27 exp Bronchiolitis/ 9939
28 bronchiolitis.ti,ab. 12722
29 1 or 2 or 3 or 4 or 5 or 6 or 7 or 8 or 9 or 10 or 11 or 12 or 13 or 14 or 15 or 16 or 17 or 18 or 19 or 20 or 21 or 22 or 23 or 24 or 25 or 26 or 27 or 28 201480
30 exp Anti-Bacterial Agents/ 820965
31 antibiotic*.ti,ab. 414149
32 30 or 31 1009816
33 reattendance.mp. 158
34 reattendance.ti,ab. 157
35 reconsultation.ti,ab. 85
36 reconsultation.mp. 88
37 further consultation.ti,ab. 169
38 further consultation.mp. 169

39	revisit$.mp.	44929
40	revisit$.ti,ab.	44867
41	repeat consult$.mp.	45
42	repeat consult$.ti,ab.	45
43	repeat visit$.mp.	412
44	repeat attend$.mp.	69
45	repeat attend$.ti,ab.	68
46	reconsult$.mp.	166
47	reconsult$.ti,ab.	153
48	reattend$.mp.	286
49	reattend$.ti,ab.	285
50	consultation rate.mp.	466
51	consultation rate.ti,ab.	466
52	consultation frequency.mp.	118
53	standardised consultation ratio.mp.	1
54	visit rate.mp.	532
55	visit frequency.mp.	606
56	attendance rate.mp.	1162
57	attendance frequency.mp.	65
58	standardised attendance ratio.mp.	0
59	return rates.mp.	752
60	future attendance.mp.	21
61	33 or 34 or 35 or 36 or 37 or 38 or 39 or 40 or 41 or 42 or 43 or 44 or 45 or 46 or 47 or 48 or 49 or 50 or 51 or 52 or 53 or 54 or 55 or 56 or 57 or 58 or 59 or 60	49689
62	29 and 32 and 61	150
63	Future Likelihood of Seeking Care.ti,ab.	1
64	Future Likelihood of Seeking Care.mp.	1
65	acute respiratory illness.mp.	1662
66	acute respiratory illness.ti,ab.	1614
67	65 or 66	1662
68	29 or 67	202616
69	61 or 63	49690
70	32 and 68 and 69	151

Embase <1974 to 2024 February 6>

1	(respiratory tract adj3 infection$).tw.	41922
2	(respiratory tract adj3 infection$).mp.	134387
3	acute pharyngitis.tw.	809
4	exp pharyngitis/	36723
5	sore throat.tw.	10928
6	exp sore throat/	25590
7	acute laryngitis.tw.	209
8	exp laryngitis/	7155
9	acute epiglottitis.tw.	673
10	exp acute epiglottitis/	501
11	acute tonsillitis.mp.	1007
12	acute tonsillitis.tw.	994
13	acute otitis externa.mp.	205
14	acute otitis externa.tw.	197
15	exp acute otitis media/	4522
16	acute otitis media.tw.	6681
17	exp acute sinusitis/	1399
18	acute sinusitis.tw.	1629
19	exp acute rhinosinusitis/	759
20	acute rhinosinusitis.tw.	884
21	exp common cold/	10069
22	common cold.tw.	5210
23	exp tracheitis/	4213
24	acute tracheitis.tw.	24
25	acute bronchitis.mp.	2287
26	acute bronchitis.tw.	2272
27	exp bronchiolitis/	26380
28	bronchiolitis.tw.	19573

29 1 or 2 or 3 or 4 or 5 or 6 or 7 or 8 or 9 or 10 or 11 or 12 or 13 or 14 or 15 or 16 or 17 or 18 or 19 or 20 or 21 or 22 or 23 or 24 or 25 or 26 or 27 or 28 235430

30 exp antibiotic agent/ 1780426

31 antibiotic$.tw. 544415

32 30 or 31 1933892

33 reattend$.mp. 500

34 reattend$.tw. 496

35 repeat visit$.mp. 718

36 repeat visit$.tw.714

37 repeat consult$.mp. 63

38 repeat consult$.tw. 63

39 repeat attend$.mp. 135

40 repeat attend$.tw. 133

41 reconsult$.mp. 215

42 reconsult$.tw. 199

43 reattendance.mp. 266

44 reattendance.tw. 262

45 reconsultation.mp. 111

46 reconsultation.tw. 105

47 further consultation.mp. 294

48 further consultation.tw. 294

49 revisit$.mp. 50017

50 revisit$.tw. 49910

51 consultation rate.mp. 568

52 consultation rate.tw. 560

53 consultation frequency.mp. 168

54 consultation frequency.tw. 162

55 standardised consultation ratio.mp. 1

56 visit rate.mp. 828

57 visit frequency.mp. 903

58 attendance rate.mp. 1824

59 attendance frequency.mp. 97

60 33 or 34 or 35 or 36 or 37 or 38 or 39 or 40 or 41 or 42 or 43 or 44 or 45 or 46 or 47 or 48 or 49 or 50 or 51 or 52 or 53 or 54 or 55 or 56 or 57 or 58 56074

61 29 and 32 and 60 238

Cochrane Library

respiratory tract adj3 infection$ OR acute pharyngitis OR sore throat OR acute epiglottitis OR laryngitis OR acute epiglottitis OR acute tonsillitis OR acute otitis media OR acute rhinitis OR acute rhinosinusitis OR common cold OR acute tracheitis OR acute bronchitis OR bronchiolitis AND antibiotic$ OR reattendance OR reconsultation AND primary care Cochrane group acute respiratory tract infections
Last search date – February 6, 2024

PubMed
((((((((((((((((((((((((respiratory tract adj3 infection$)) OR (acute pharyngitis)) OR (sore throat)) OR (acute epiglottitis)) OR (laryngitis)) OR (acute epiglottitis)) OR (acute tonsillitis)) OR (acute otitis media)) OR (acute rhinitis)) OR (acute rhinosinusitis)) OR (common cold)) OR (acute tracheitis)) OR (acute bronchitis)) OR (bronchiolitis)) AND (antibiotic$)) OR (reattendance)) OR (reconsultation)) AND (primary care)
Filters: Observational Study, Randomized Controlled Trial
Last search date – February 6, 2024

Web of Science February,6 2024

1	ALL=(ANTIBIOTIC) OR ALL=(antibACTERIAL) OR ALL=(ANTIMICROBIAL)	830559
2	ALL=(randomised) OR ALL=(randomized) AND ALL=(randomised) AND ALL=(controlled) AND ALL=(TRIAL)	991277
3	ALL=(respiratory infection) OR ALL=(upper respiratory tract infection) OR ALL=(urti) OR ALL=(sore throat) OR ALL=(throat infection) OR ALL=(pharyngitis) OR ALL=(tonsillitis) OR ALL=(acute otitis media) OR ALL=(sinusitis) OR ALL=(coryza) OR ALL=(common cold) OR ALL=(cough) OR ALL=(lower respiratory tract) OR ALL=(bronchitis) OR ALL=(laryngitis) OR ALL=(laryngotracheitis) OR ALL=(trachietis) OR ALL=(bronchiolitis) AND ALL=(rhinitis)	305925
4	ALL=(primary health care) OR ALL=(primary care) OR ALL=(general practice) OR ALL=(family medicine)	1418979
5	ALL=(reattendance) OR ALL=(reattend) OR ALL=(reconsultation) OR ALL=(further consultation) OR ALL=(future consultation) OR ALL=(repeat consultation) OR ALL=(further attendance) OR ALL=(future attendance) OR ALL=(repeat attendance) OR ALL=(revisit) OR ALL=(further visit) OR ALL=(repeat visit) OR ALL=(future visit) OR ALL=(reconsult) OR ALL=(consultation rate) OR ALL=(consultation frequency) OR ALL=(visit rate) OR ALL=(visit frequency) OR ALL=(attendance rate) OR ALL=(attendance frequency)	294806

	OR ALL=(standardised attendance ratio)	
6	#1 AND #2 AND #3 AND #4 AND #5	251

Clinicaltrials.gov
Acute Respiratory Infections OR Respiratory Tract Infections AND antibiotic prescription
Filters: Interventional, All Ages
Last search date – February 6, 2024

Clinicaltrialsregister.eu
Respiratory tract infection AND antibiotic AND randomized OR placebo-controlled AND adult OR paediatric OR child
Last search date – February 6, 2024
