# Supplementary material for: Effects of antibiotic prescribing for respiratory tract infection on future consultations in primary care: a systematic review and meta-analysis
Source: BMJ Open. 2025 Jul 28;15(7):e099357. doi: 10.1136/bmjopen-2025-099357 (PMC12306365; doi:10.1136/bmjopen-2025-099357)

Supplementary tables & figures

Supplementary Table 1: Medline search strategy (Ovid MEDLINE® ALL <1946 to 6th Feb 2024>)

N	Search term	Results
1	(respiratory tract adj3 infection$).ti,ab.	27723
2	(respiratory tract adj3 infection$).mp. [mp=title, book title, abstract, original title, name of substance word, subject heading word, floating sub-heading word, keyword heading word, organism supplementary concept word, protocol supplementary concept word, rare disease supplementary concept word, unique identifier, synonyms, population supplementary concept word, anatomy supplementary concept word]	59888
3	exp Pharyngitis/	16725
4	acute pharyngitis.ti,ab.	630
5	exp Laryngitis/	4119
6	acute laryngitis.ti,ab.	198
7	sore throat.mp. or Pharyngitis/	13662
8	sore throat.ti,ab.	6965
9	exp Epiglottitis/	1053
10	acute epiglottitis.ti,ab.	617
11	exp Tonsillitis/	8247
12	acute tonsillitis.ti,ab.	773
13	exp Otitis Media/	25752
14	acute otitis media.ti,ab.	5436
15	exp Otitis Externa/	2651
16	acute otitis externa.ti,ab.	158
17	exp Sinusitis/	23177
18	acute sinusitis.ti,ab.	1202
19	exp Rhinitis/ or exp Sinusitis/	53209
20	acute rhinosinusitis.ti,ab.	672
21	exp Common Cold/	4427
22	common cold.ti,ab.	4229
23	exp Tracheitis/	1560
24	acute tracheitis.ti,ab.	13
25	exp Bronchitis/	31453
26	acute bronchitis.ti,ab.	1532
27	exp Bronchiolitis/	9835
28	bronchiolitis.ti,ab.	12586
29	1 or 2 or 3 or 4 or 5 or 6 or 7 or 8 or 9 or 10 or 11 or 12 or 13 or 14 or 15 or 16 or 17 or 18 or 19 or 20 or 21 or 22 or 23 or 24 or 25 or 26 or 27 or 28	200078
30	exp Anti-Bacterial Agents/	814589
31	antibiotic*.ti,ab.	407211
32	30 or 31	999639
33	reattendance.mp.	153
34	reattendance.ti,ab.	152
35	reconsultation.ti,ab.	82
36	reconsultation.mp.	85
37	further consultation.ti,ab.	169
38	further consultation.mp.	169
39	revisit$.mp.	44241

N	Search term	Results
40	revisit$.ti,ab.	44179
41	repeat consult$.mp.	43
42	repeat consult$.ti,ab.	43
43	repeat visit$.mp.	405
44	repeat attend$.mp.	65
45	repeat attend$.ti,ab.	64
46	reconsult$.mp.	156
47	reconsult$.ti,ab.	143
48	reattend$.mp.	281
49	reattend$.ti,ab.	280
50	consultation rate.mp.	461
51	consultation rate.ti,ab.	461
52	consultation frequency.mp.	116
53	standardised consultation ratio.mp.	1
54	visit rate.mp.	524
55	visit frequency.mp.	586
56	attendance rate.mp.	1142
57	attendance frequency.mp.	63
58	standardised attendance ratio.mp.	0
59	return rates.mp.	741
60	future attendance.mp.	21
61	33 or 34 or 35 or 36 or 37 or 38 or 39 or 40 or 41 or 42 or 43 or 44 or 45 or 46 or 47 or 48 or 49 or 50 or 51 or 52 or 53 or 54 or 55 or 56 or 57 or 58 or 59 or 60	48907
62	29 and 32 and 61	147
63	Future Likelihood of Seeking care.ti,ab.	1
64	Future Likelihood of Seeking care.mp.	1
65	acute respiratory illness.mp.	1637
66	acute respiratory illness.ti,ab.	1593
67	65 or 66	1637
68	29 or 67	201196
69	61 or 63	48908
70	32 and 68 and 69	148

Supplementary Table 2: Embase search strategy (Embase <1974 to 6th Feb 2024>)

N	Search Term
1	(respiratory tract adj3 infection$).tw.
2	(respiratory tract adj3 infection$).mp.
3	acute pharyngitis.tw.
4	exp pharyngitis/
5	sore throat.tw.
6	exp sore throat/
7	acute laryngitis.tw.
8	exp laryngitis/
9	acute epiglottitis.tw.
10	exp acute epiglottitis/
11	acute tonsillitis.mp.
12	acute tonsillitis.tw.
13	acute otitis externa.mp.
14	acute otitis externa.tw.
15	exp acute otitis media/
16	acute otitis media.tw.
17	exp acute sinusitis/
18	acute sinusitis.tw.
19	exp acute rhinosinusitis/
20	acute rhinosinusitis.tw.
21	exp common cold/
22	common cold.tw.
23	exp tracheitis/
24	acute tracheitis.tw.
25	acute bronchitis.mp.
26	acute bronchitis.tw.
27	exp bronchiolitis/
28	bronchiolitis.tw.
29	1 or 2 or 3 or 4 or 5 or 6 or 7 or 8 or 9 or 10 or 11 or 12 or 13 or 14 or 15 or 16 or 17 or 18 or 19 or 20 or 21 or 22 or 23 or 24 or 25 or 26 or 27 or 28
30	exp antibiotic agent/
31	antibiotic$.tw.
32	30 or 31
33	reattend$.mp.
34	reattend$.tw.
35	repeat visit$.mp.
36	repeat visit$.tw.
37	repeat consult$.mp.
38	repeat consult$.tw.
39	repeat attend$.mp.
40	repeat attend$.tw.
41	reconsult$.mp.
42	reconsult$.tw.
43	reattendance.mp.
44	reattendance.tw.
45	reconsultation.mp.
46	reconsultation.tw.
47	further consultation.mp.
48	further consultation.tw.
49	revisit$.mp.
50	revisit$.tw.

N	Search Term
51	consultation rate.mp.
52	consultation rate.tw.
53	consultation frequency.mp.
54	consultation frequency.tw.
55	standardised consultation ratio.mp.
56	visit rate.mp.
57	visit frequency.mp.
58	attendance rate.mp.
59	attendance frequency.mp.
60	33 or 34 or 35 or 36 or 37 or 38 or 39 or 40 or 41 or 42 or 43 or 44 or 45 or 46 or 47 or 48 or 49 or 50 or 51 or 52 or 53 or 54 or 55 or 56 or 57 or 58
61	29 and 32 and 60

Supplementary Table 3: Search strategy for Cochrane Central

COCHRANE CENTRAL 4 MAY 23 2023
respiratory tract adj3 infection$ OR acute pharyngitis OR sore throat OR acute epiglottitis OR laryngitis OR acute epiglottitis OR acute tonsillitis OR acute otitis media OR acute rhinitis OR acute rhinosinusitis OR common cold OR acute tracheitis OR acute bronchitis OR bronchiolitis AND antibiotic$ OR reattendance OR reconsultation AND primary care

Supplementary Table 4: Search strategy for Pubmed (MEDLINE)

Pubmed 4th May 2023
((((((((((((((((((((((((respiratory tract adj3 infection$)) OR (acute pharyngitis)) OR (sore throat)) OR (acute epiglottitis)) OR (laryngitis)) OR (acute epiglottitis)) OR (acute tonsillitis)) OR (acute otitis media)) OR (acute rhinitis)) OR (acute rhinosinusitis)) OR (common cold)) OR (acute tracheitis)) OR (acute bronchitis)) OR (bronchiolitis)) AND (antibiotic$)) OR (reattendance)) OR (reconsultation)) AND (primary care)
Filters: Observational Study, Randomized Controlled Trial

Supplementary Table 5: Search in Web of Science

Science Citation Index Expanded – 1900 to present

Conference proceedings citation index – Science – 1990 to present

#1 AND #2 AND #3 AND #4 AND #5

Query #1	ALL=(ANTIBIOTIC) OR ALL=(antibACTERIAL) OR ALL=(ANTIMICROBIAL)
Query #2	**ALL=(randomised controlled) OR ALL=(randomized controlled) OR ALL=(clinical trial) OR ALL=(randomized trial) OR ALL=(randomised trial)**
Query #3	ALL=(respiratory infection) OR ALL=(upper respiratory tract infection) OR ALL=(urti) OR ALL=(sore throat) OR ALL=(throat infection) OR ALL=(pharyngitis) OR ALL=(tonsillitis) OR ALL=(acute otitis media) OR ALL=(sinusitis) OR ALL=(coryza) OR ALL=(common cold) OR ALL=(cough) OR ALL=(lower respiratory tract) OR ALL=(bronchitis) OR ALL=(laryngitis) OR ALL=(laryngotracheitis) OR ALL=(trachietis) OR ALL=(bronchiolitis) AND ALL=(rhinitis)
Query #4	ALL=(primary health care) OR ALL=(primary care) OR ALL=(general practice) OR ALL=(family medicine)
Query #5	ALL=(reattendance) OR ALL=(reattend) OR ALL=(reconsultation) OR ALL=(further consultation) OR ALL=(future consultation) OR ALL=(repeat consultation) OR ALL=(further attendance) OR ALL=(future attendance) OR ALL=(repeat attendance) OR ALL=(revisit) OR ALL=(further visit) OR ALL=(repeat visit) OR ALL=(future visit) OR ALL=(reconsult) OR ALL=(consultation rate) OR ALL=(consultation frequency) OR ALL=(visit rate) OR ALL=(visit frequency) OR ALL=(attendance rate) OR ALL=(attendance frequency) OR ALL=(standardised attendance ratio)

This search produced 277 hits.

Link to search results here: https://www.webofscience.com/wos/woscc/summary/6a96f376-2986-4c41-a953-75664a5cdaa1-cb0216f5/relevance/1

Supplementary Table 6: General information & selection criteria

Study title		
Study ID (surname of first author + year of publication)		
ID of person extracting data (Initials)		
Study author's contact details		
Publication type (full report or abstract)		
Date form was completed (*dd/mm/yyyy*)		
Study characteristics	Eligibility criteria	Eligibility criteria met.<br>(Yes) (No) (Unclear)
Study design	Randomised controlled trial (unblinded)<br>Cohort study	
Population	Adults or children with respiratory tract infections presenting in primary care	
Intervention (exposure)	Antibiotics (immediate or delayed)	
Comparison	No antibiotics	
Outcome	Future reattendance: ≥28 days / 1 month	
Setting	Primary care	

Include<br>(Tick as appropriate)	Exclude<br>(Tick as appropriate)
Reasons for exclusion if applicable	

Supplementary Table 7: Characteristics of included studies

	Description as stated in the report or paper	Location in the text
Aim of the study		
Study design		
Start date		
End date		
Study duration		
Sample size		
Country of origin		
Ethical approval needed/obtained for the study		
Characteristics of the population		
Inclusion criteria		
Exclusion criteria		
Type of intervention (immediate or delayed antibiotic prescription)		
Information on the outcome (estimate measure, unit of measurement, dichotomous or continuous variable)		
Source of funding		
Conflict of interest		

Supplementary Figure 1: Forest plot of relative risk of reattendance for antibiotics versus no antibiotics, RCTs and cohort studies excluding Williamson, 2006

Study identifier, year	Antibiotic arm: events	Antibiotic arm: total	Control arm: events	Control arm: total	Relative risk (95% CI)	% Weight
RCT studies: Immediate antibiotics						
Little, 1997	106	222	78	192	1.18 (0.94, 1.46)	22.03
Moore, 2009	38	183	37	204	1.14 (0.76, 1.72)	6.43
Subgroup (I-squared = 0.0%)					1.17 (0.96, 1.42)	28.45
RCT studies: Delayed antibiotics						
Little, 1997	68	182	78	192	0.92 (0.71, 1.19)	16.42
Little, 2014 a	42	107	39	122	1.23 (0.87, 1.74)	8.66
Little, 2014 b	45	114	39	122	1.23 (0.87, 1.74)	8.94
Little, 2014 c	33	105	39	122	0.98 (0.67, 1.44)	7.23
Little, 2014 d	39	106	39	122	1.15 (0.80, 1.65)	8.21
Moore, 2009	37	188	37	204	1.09 (0.72, 1.64)	6.30
Subgroup (I-squared = 0.0%)					1.07 (0.93, 1.23)	55.76
Cohort studies:						
Herz, 1988	24	84	14	88	1.80 (1.00, 3.23)	3.08
Little, 2014	136	326	39	122	1.38 (1.01, 1.78)	12.71
Subgroup (I-squared = 0.0%)					1.39 (1.07, 1.80)	15.79
Heterogeneity between groups: p = 0.215						
Overall (I-squared = 0.0%)					1.14 (1.03, 1.27)	100.00

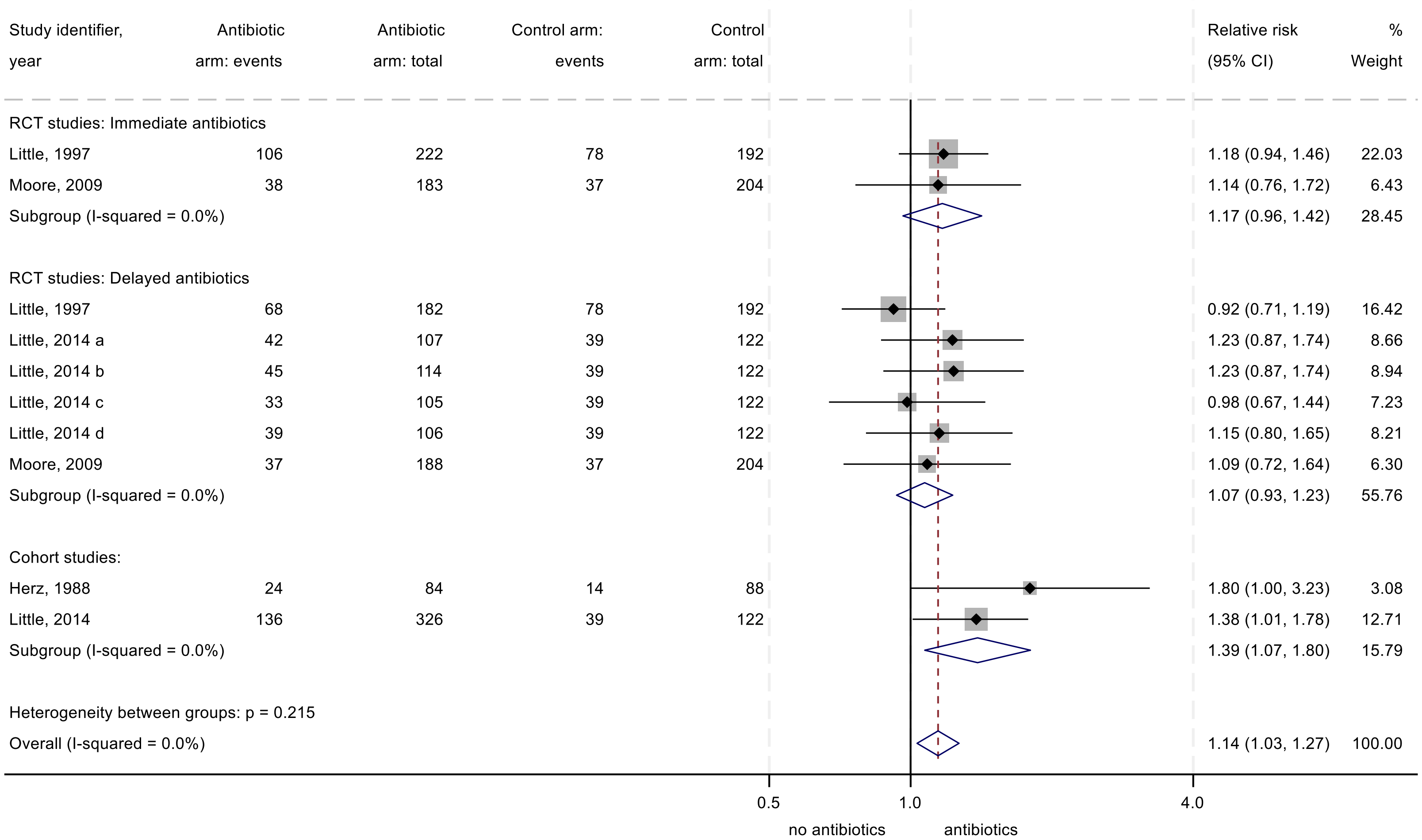

Supplementary Figure 2: Forest plot of relative risk of reattendance for antibiotics versus no antibiotics, cohort studies excluding Williamson, 2006

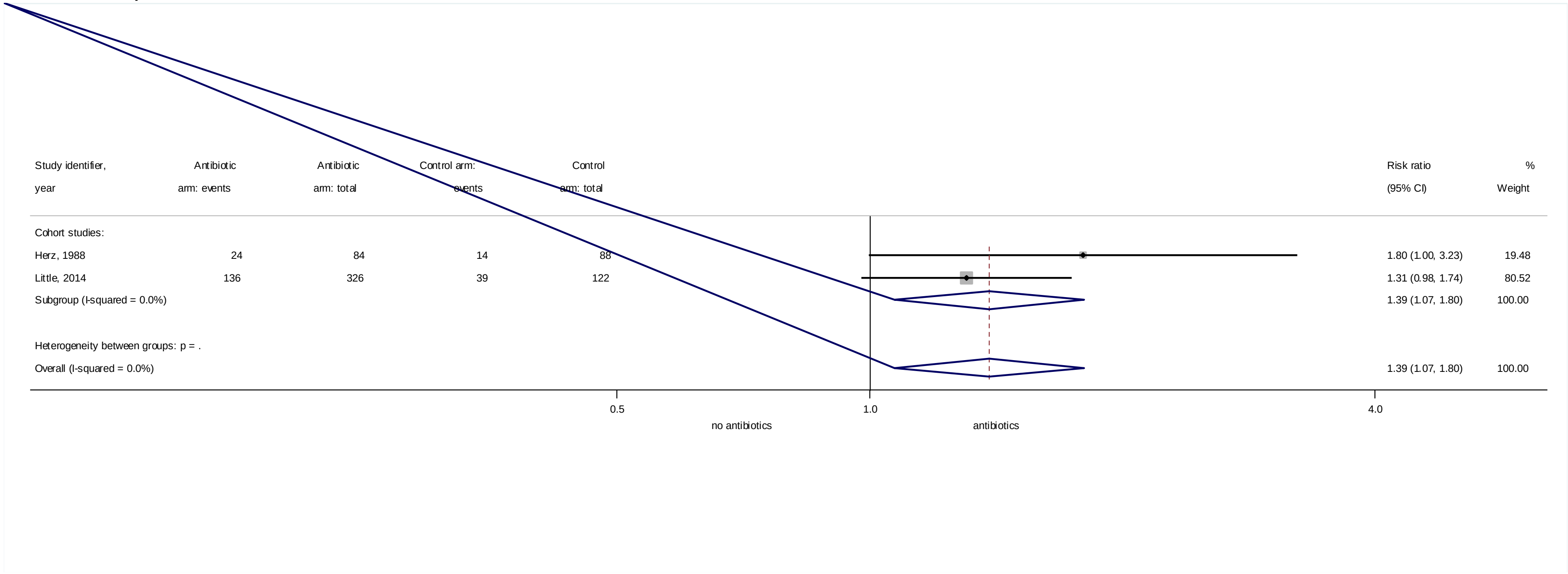

Supplement: online supplemental file 2 [file bmjopen-15-7-s002.pdf]
