# Supplementary material for: Effects of antibiotic prescribing for respiratory tract infection on future consultations in primary care: a systematic review and meta-analysis
Source: BMJ Open. 2025 Jul 28;15(7):e099357. doi: 10.1136/bmjopen-2025-099357 (PMC12306365; doi:10.1136/bmjopen-2025-099357)
Supplement: online supplemental file 3 [file bmjopen-15-7-s003.pdf]

sensitivity analysis

Excluded Study	Pooled AUC (95% CI)	I^2	p (Q-test)
tan 2017	0.73(0.67, 0.79)	42.2%	0.140
Liu 2018	0.70(0.67, 0.73)	9.9%	0.350
Cai 2020	0.73(0.68, 0.79)	45.3%	0.120
Werba 2021	0.77(0.71, 0.83)	0.0%	0.962
Xu 2022	0.72(0.67, 0.78)	35.0%	0.188
Li 2023	0.71(0.67, 0.76)	17.1%	0.306
