# Supplementary material for: Effects of antibiotic prescribing for respiratory tract infection on future consultations in primary care: a systematic review and meta-analysis
Source: BMJ Open. 2025 Jul 28;15(7):e099357. doi: 10.1136/bmjopen-2025-099357 (PMC12306365; doi:10.1136/bmjopen-2025-099357)
Supplement: Supplementary file 4 [file bmjopen-2025-099357.reviewer_comments.pdf]

PEER REVIEW HISTORY

BMJ Open publishes all reviews undertaken for accepted manuscripts. Reviewers are asked to complete a checklist review form and are provided with free text boxes to elaborate on their assessment. These free text comments are reproduced below.

ARTICLE DETAILS

Title (Provisional)

The effects of antibiotic prescribing for respiratory tract infection on future consultations in primary care: a systematic review and meta-analysis

Authors

Adamu, Ibrahim; Lambert, Amanda; Bello, Safiyya; Abdulmalik, Fatima Aminu; Marshall, Tom

---

VERSION 1 - REVIEW

**Reviewer**	**1**
**Name**	**Lee, Joseph**
**Affiliation**	**University of Oxford, Nuffield Department of Primary Care Health Sciences**
**Date**	**12-Feb-2025**
**COI**	**None**

Describes a systematic review of antibiotics for RTI with reattendance as the outcome of interest.This is an important angle, and clinically interesting. I just can't be sure the analysis is quite right.

Looking at "figure 2 Forest plot of RR of reattendance for antibiotics versus no antibiotics, RCTs" and the methods, I was not able to tell how the different arms of Little 2014 have been analysed - it looks like the control group has been used four times? Cochrane recommends combining the intervention arms, or splitting the control arm (https://training.cochrane.org/sites/training.cochrane.org/files/public/uploads/resources/embedded_resources/gomo/cesm/tutorials/multi-arm-studies/index.htm). I suspect this would reduce the precision of the pooled estimate, but it might not. It looks like the same thing has happened with Little 97 and Moore 2009, only the arms are in different subgroups with the control double included in each subgroup?

I see there is a bit in the results about testing non-independence (suggest move to methods) but is this really this issue? Isn't it about double (and triple) counting?

I could be that this analysis is all fine, but if so please add a simple explanation to help the reader understand it.

**Reviewer**	**2**
**Name**	**King, David**
**Affiliation**	**University of Queensland, Faculty of Medicine**
**Date**	**06-May-2025**
**COI**	**None**

Just a few minor points and suggestions.

In the Results section, p.8, you mention one reason for exclusion of studies being "reported recurrent episodes of URTI rather than reattendances" - while correct, it took me a while to understand the comparison. May be less ambiguous to say something such as "reported recurrent episodes rather than reattandences for URTI".

The last sentence on this page - the number should come before rather than after the word 'participants' - ie. ..209,138 participants...

The Williamson (2006) study Table 1 mentions the outcome as reattendance with OAM at any time, yet your inclusion criteria set primary outcome of reattendance at least 28 days later. You might want to explain or justify this seeming descrepancy in the text somewhere, or * it as a footnote under the table.

You might also consider adding to your discussion that a single episode of prescribing vs not prescribing may be insufficient for breaking expectations and knowledge of patient in effectiveness and thus presentations for antibiotics for colds, and a study looking at the accumulative benefit of resisting unnecessary antibiotic usage may actually be higher than detected in these 'single' intervention design studies.

**Reviewer**	**3**
**Name**	**Dack, Kyle**
**Affiliation**	**University of Bristol, MRC Integrative Epidemiology Unit**
**Date**	**28-May-2025**
**COI**	**None**

Statistical review summary:

Although this meta-analysis includes only 5 papers, it applies valid methods and interprets the results clearly and with suitable caution. My only major concern is that there are some

details missing from the statistical analysis section about the meta-analyses, such as the types performed and parameters used.

Full comments:

1. Abstract: The abstract states that cohort studies were meta-analysed separately, but in the methods and results, there is also a meta analysis of RCTs + cohort studies.

Methods:

2. Databases searched: full queries are provided in supplementary tables. However, it would help readers to provide a high-level summary of the search strategy, such as the main keywords it was built around.

3. Risk of bias: the NOS variants were used for observational studies. Most types of NOS have a criteria for comparability, which requires authors to decide what are the key confounders which must be adjusted for a study to meet the criteria. I suggest stating what studies needed to do to meet this particular criteria to ensure the RoB is fully transparent.

4. The meta-analysis explanation is a little unclear: which software and specific functions were used to perform it? In the results, it is stated that a random-effects meta-analysis was performed – this should also be part of the statistical analysis section, including to explain why this was chosen over standard fixed effects.

5. The statistical analysis section states that there were two meta-analyses performed, with the secondary meta-analyses being RCTs + cohort studies. How were these made comparable?

Results

6. Figure 1. I think this would look a lot nicer if boxes were added for the exclusion reasons – there may be a PRISMA flowchart template available online which contains this.

7. Little 2014: when it is first mentioned that this paper includes both an RCT and cohort study, I suggest a brief clarification that the cohort study included a subset of patients from the RCT (if my interpretation is correct). This is explained in the "cohort studies" section but readers will likely be querying this the first time it is mentioned at the beginning of the results.

8. The meta-analysis section identifies that the control group was the same in the 3 RCTs. I agree with the approach the authors have taken and their interpretation, but this part of the investigation should also be stated in the methods – three-level random effects meta-analysis should be reported along with the details of the parameters used.

9. "Random effects meta-analysis of both RCTs and cohort studies found a combined RR of 1.10 (95%CI: 1.08 to 1.11)." This random effects model seems to include Little 2014 twice, using both the RCT results and the cohort study results (figure 4), but these were not independent and used some of the same patients. This should be justified in the methods

and perhaps a similar investigation to the RCT meta-analysis performed to assess the validity of these comparisons.

10. A third meta-analysis is reported, of cohort studies alone, but this is missing from the methods.

11. Figures 2-4. I am unsure of the x-axis on these forest plots. The plots show the RR of reattendance, but the x-axis is labelled “antibiotics” and “no antibiotics”, which gives the impression this is showing the RR of antibiotic use. I think it should be “reattendance” and “non-reattendance”?

VERSION 1 - AUTHOR RESPONSE

Reviewer: 1

Dr. Joseph Lee, University of Oxford

Asks for clarification about how the different arms of Little 2014 (which has one control group and four intervention arms) have been included in the meta-analysis. Points out that Cochrane guidance recommends combining the intervention arms, or splitting the control arm.

Thank you for your comment. The four intervention arms of Little 2014 have been included with the whole control group. Although the resulting relative risks will be correlated as the same participants are included within each control group, three-level random effects meta-analysis has been used to investigate the size of the effect of doing this. Results from the three-level analysis were in fact very similar to the standard random effects meta-analysis.

Asks if the same thing has happened with Little 1997 and Moore 2009, only the arms are in different subgroups with the control double included in each subgroup?

Little 1997 and Moore 2009 also include the same control group within each of the RCT subgroups.

Suggests moving the section on testing non-independence to the Methods. The reviewer also comments that it could be that this analysis is all fine, but if that a simple explanation should be added to help the reader understand it.

The Methods section now includes a statement on the three-level meta-analysis and its use for non-independence of control groups.

Reviewer: 2

Dr. David King, University of Queensland

Just a few minor points and suggestions.

Suggests rephrasing the line on reasons for exclusion in the Results section, p.8, from "reported recurrent episodes of URTI rather than reattendances" to "reported recurrent episodes rather than reattendences for URTI".

Thank you for pointing this out. This has been rephrased.

Suggests that in the last sentence on page 9 the number should come before rather than after the word 'participants' - ie. ..209,138 participants...

Thank you for pointing this out. This has been corrected.

The Williamson (2006) study Table 1 mentions the outcome as reattendance with OAM at any time in the future, yet your inclusion criteria set primary outcome of reattendance at least 28 days later. This discrepancy should be commented on in the text as a footnote to Table 1.

Thank you for pointing this out. A sentence has been added in the text and a footnote to Table 1.

Suggests that adding to the Discussion that a single episode of prescribing or not prescribing may be insufficient to change a patient's expectations in relation to effectiveness of antibiotics and thus change presentations for antibiotics for colds. A study looking at the accumulative benefit of resisting unnecessary antibiotic usage may actually be higher than detected in these 'single' intervention design studies.

A sentence has been added to the discussion to reflect the possibility that repeated prescribing (or non-prescribing) may have a cumulatively larger effect on future reattendance.

Reviewer: 3

Dr. Kyle Dack, University of Bristol (statistical review)

Although this meta-analysis includes only 5 papers, it applies valid methods and interprets the results clearly and with suitable caution. My only major concern is that there are some details missing from the statistical analysis section about the meta-analyses, such as the types performed and parameters used.

Abstract:

The abstract states that cohort studies were meta-analysed separately, but in the methods and results, there is also a meta-analysis of RCTs + cohort studies.

Thank you for pointing this out. The Abstract has been rewritten and the third meta-analysis has now been mentioned.

Methods:

Databases searched: full queries are provided in supplementary tables. However, it would help readers to provide a high-level summary of the search strategy, such as the main keywords it was built around.

We have added a short sentence to the Methods explaining that the search was constructed around synonyms for upper respiratory tract infections and synonyms for reattendance.

Risk of bias:

The Newcastle Ottawa Scale has a criterion for comparability, which requires authors to decide what are the key confounders which should be included in adjustment. The reviewer suggests stating what studies needed to do to meet the NOS comparability criterion.

Thank you for your feedback. In line with the Newcastle-Ottawa Scale (NOS) guidance, comparability assesses whether the exposed and unexposed groups are similar in terms of key confounding variables, either through design matching or analytical adjustment. For this review, studies were awarded up to two stars in the comparability domain based on the following criteria:

One star was awarded if the analysis adjusted for age, which was considered a key confounder given its strong association with both antibiotic prescribing patterns and health outcomes such as reconsultation or complications.

A second star was awarded if the study additionally adjusted for at least one other relevant confounder, such as sex, socioeconomic status, comorbidity, prior healthcare use etc.

Accordingly:

- *Williamson 2006 scored two stars, as it adjusted for age, sex, multiple deprivation index, ENT referral, and practice prescribing patterns, even though baseline characteristics were not tabulated.*
- *Herz 1988 scored no stars, as there was no baseline comparison table and no adjustment for confounders in the analysis. A statement that groups were "demographically similar" was not sufficient per NOS guidance.*
- *Little 2014 also scored two stars, having reported baseline differences and adjusted for previous infections, sex, age, diagnosis, follow-up time, baseline symptom severity, dosing, steam, and smoking in multivariable analyses.*

A statement explaining this has been added in the result section.

The meta-analysis explanation is a little unclear: which software and specific functions were used to perform it? In the results, it is stated that a random-effects meta-analysis was performed – this should also be part of the statistical analysis section, including to explain why this was chosen over standard fixed effects.

We have now stated in the Meta-analysis section in the Methods that Stata 18 package admetan was used and added a sentence to explain the use of random effects meta-analysis. Stata package meta multilevel was used for three-level random effects meta-analysis.

The statistical analysis section states that there were two meta-analyses performed, with the secondary meta-analyses being RCTs + cohort studies. How were these made comparable?

There are described methods for combining RCTs and cohort studies in the same meta-analyses. This is described in Scenario 2 of Sarri 2022, which refers to the case when inference is from both RCTs and observational studies

Reference: Sarri G, Patorno E, Yuan H, et al Framework for the synthesis of non-randomised studies and randomised controlled trials: a guidance on conducting a systematic review and meta-analysis for healthcare decision making BMJ Evidence-Based Medicine 2022;27:109-119.

Results

Figure 1. I think this would look a lot nicer if boxes were added for the exclusion reasons – there may be a PRISMA flowchart template available online which contains this.

Thank you for this suggestion. Boxes have been added to the exclusion reasons

Little 2014: when it is first mentioned that this paper includes both an RCT and cohort study, I suggest a brief clarification that the cohort study included a subset of patients from the RCT (if my interpretation is correct). This is explained in the “cohort studies” section but readers will likely be querying this the first time it is mentioned at the beginning of the results.

Thank you for pointing this out. This has been explained more clearly in the Results.

The meta-analysis section identifies that the control group was the same in the 3 RCTs. I agree with the approach the authors have taken and their interpretation, but this part of the investigation should also be stated in the methods – three-level random effects meta-analysis should be reported along with the details of the parameters used.

Thank you. Two sentences have been added to the Methods to reflect this.

“Random effects meta-analysis of both RCTs and cohort studies found a combined RR of 1.10 (95%CI: 1.08 to 1.11).” This random-effects model seems to include Little 2014 twice, using both the RCT results and the cohort study results (figure 4), but these were not independent and used some of the same patients. This should be justified in the methods and perhaps a similar investigation to the RCT meta-analysis performed to assess the validity of these comparisons.

Thank you. The non-independence has now been accounted for in the methods (see sentences added mentioned in point 20). The three-level meta-analysis already presented in the results

referred to the analysis that included both RCTs and cohort studies. Results from the three-level analysis that just included the RCTs has now been added.

A third meta-analysis is reported, of cohort studies alone, but this is missing from the methods.

This was pointed out by other reviewers and the third meta-analysis has now been added to the Methods.

Figures 2-4. I am unsure of the x-axis on these forest plots. The plots show the RR of reattendance, but the x-axis is labelled "antibiotics" and "no antibiotics", which gives the impression this is showing the RR of antibiotic use. I think it should be "reattendance" and "non-reattendance"?

The figures have been relabelled to show the x-axis as "no reattendance" and "reattendance".

VERSION 2 - REVIEW

**Reviewer**	**1**
**Name**	**Lee, Joseph**
**Affiliation**	**University of Oxford, Nuffield Department of Primary Care Health Sciences**
**Date**	**09-Jul-2025**
**COI**	

All comments have been answered.
